# Developing and psychometrics assessment of a checklist for safe intrahospital patient transfer

**DOI:** 10.1186/s12913-025-13987-w

**Published:** 2026-01-08

**Authors:** Mahdie Saberi, Mohammad-Ehsan Adib, Mohsen Adib-Hajbaghery, Mohammad Mahdi Heidari, Atefe Fallah

**Affiliations:** 1 Trauma Nursing Research Center, Kashan University of Medical Sciences, Kashan, Iran; 2 Kashan University of Medical Sciences, Kashan, Iran; 3 Infectious Diseases Research Center, Kashan University of Medical Sciences, Kashan, Iran

**Keywords:** Patient safety, Patient transfer, Intrahospital, Nurses

## Abstract

**Background:**

The absence of clear policies and standardized guidelines for intrahospital transfer poses a substantial threat to patient safety. To address this gap, we designed a study to develop a comprehensive, standardized checklist for safe intrahospital patient transfer within Iranian hospitals.

**Methods:**

A sequential exploratory mixed-methods design was employed in Shahid-Beheshti hospital, Kashan, Iran, in 2023 to develop and validate a checklist for safe intrahospital patient transfer. The process was planned and conducted in two phases: qualitative item generation and psychometric evaluation. Phase I involved item generation through a qualitative content analysis of the literature to identify essential care measures (across the pre-transfer, intra-transfer, and post-transfer phases) and expert consultations to elicit the experts’ tacit professional knowledge on the phenomenone under the study. Then, the research team integrated items generated by the two aforementioned methods and created a preliminary item pool. The research team then synthesized and refined these expert-generated items with those derived from the literature to create a comprehensive draft checklist. The validation process included assessments of face validity, content validity, and construct validity. To evaluate reliability, inter-rater agreement, Cohen’s kappa, intraclass correlation coefficients (ICC), and internal consistency (Cronbach’s alpha) were used.

**Results:**

The initial item pool consisted of 55 items, which were subsequently reduced to 45 items during the face validity and qualitative content validity assessments. Then, the number of items was reduced to 38 during the content validity ratio (CVR) and content validity index (CVI) assessments. The CVR of the remaining items ranged from 0.9 to 0.98, while their CVI ranged from 0.9 to 0.99. The mean Cohen’s kappa across items was 0.62 ± 0.12. The ICC for the total score was 0.86. Cronbach’s alpha for the entire checklist was 0.79. In construct validity assessment, three factors, consistent with the three domains of the checklist, were extracted that cumulatively explained 46.79% of the variance in patient transfer safety.

**Conclusion:**

A comprehensive intrahospital patient transfer checklist was developed, demonstrating good validity and reliability, and can be used by healthcare professionals, particularly nurses, to ensure safe intrahospital patient transfers.

**Supplementary Information:**

The online version contains supplementary material available at 10.1186/s12913-025-13987-w.

## Background

Patient safety is a crucial aspect of healthcare quality [1]. According to the World Health Organization (WHO), patient safety involves preventing errors and adverse events during healthcare delivery [2]. Intrahospital transfers (IHTs) are common procedures performed for diagnostic or therapeutic purposes [3]. However, unsafe IHTs pose a significant threat to patient safety [4, 5].

Safe patient transfer involves several important components, including: deciding to transfer, ensuring effective communication between the originating and destination units and between the transfer team and the patient, stabilizing and preparing the patient prior to transfer, selecting the appropriate mode of transfer, providing the necessary personnel and equipment to accompany the patient, monitoring the patient throughout the transfer, and accurately documenting and handing over the patient to the destination unit. Careful attention to these components can help prevent adverse events during hospital transfers [6, 7].

Research indicates that patient transfer rates vary widely across hospitals and also the global incidence of adverse events during IHTs varies widely, ranging from 3% to 75% [8]. Studies from Iran report that over 50% of patient transfers are suboptimal, poor in quality, and do not adhere to standard protocols [9, 10]. Non-standard patient transfers can result in serious adverse events such as falls, airway complications, hypothermia, hemodynamic disturbances, blood pressure fluctuations, shock, increased intracranial pressure, and even patient death. Such outcomes may lead to legal issues for healthcare staff, increased medical costs, and reduced patient satisfaction with the healthcare system [11, 12].

Safe patient transfer can be compromised by various factors, such as equipment failures, staff shortages, and environmental issues [13–15]. Key treatment team-related causes are insufficient personnel, lack of experience, inadequate knowledge among staff accompanying patients during transfer, poor communication within the medical team, malfunctioning equipment and monitors, and depletion of portable oxygen [14, 16]. Common patient-related factors also include restlessness, deterioration, and instability of physiological condition [9]. Some studies have identified the training of medical staff, especially nurses, as key prerequisites for ensuring standard and safe IHTs [17]. However, education alone may not suffice without a well-established and approved guideline for these transfers. The absence of clear policies and standardized guidelines has been cited as the primary reason for non-standard IHTs [16]. Consequently, some researchers have developed guidelines or checklists for this procedure [5, 10, 18]. Although these checklists have effectively improved the quality of intrahospital patient transfers [19–21], their use has been limited for two main reasons. First, many of these checklists have mainly focused on the transport of pediatric surgery [18] or critically ill patients [5, 19, 20] or those in intensive care units [10]. Therefore, the safety and quality of transferring patients to and from general wards, or those who perceived relatively stable but remain vulnerable, have been largely overlooked. Second, the development of these tools is often context-specific, influenced by local resources, staffing models, and institutional policies. This limits their generalizability to diverse healthcare settings, particularly in settings like Iran, where systemic factors such as staff shortages and training gaps may be more pronounced. Hence, it is imperative to develop a comprehensive contextually adapted, standardized guideline that addresses the safety of IHTs for all patients, including those who appear relatively stable. This guideline should be integrated into training programs for all healthcare staff, particularly nurses, to increase the quality of IHTs and reduce the incidence of adverse events.

A review of the literature reveals that there are no comprehensive standards for safe patient transfers in Iran. Additionally, Iranian nursing curricula lack content on safe intrahospital patient transfer, and medical staff, particularly nurses, receive no specialized trained in this area. Adib-HajBagheri (2012) conducted the first study addressing this gap in Iran, highlighting deficiencies in both the formal nursing curriculum and in-service training programs for nurses [13]. Similarly, Alamanou and Brokalaki (2014) highlighted the critical role of nurses in IHTs and emphasized the need for clear policies to guide this process [15].

Due to the variation in healthcare system structures, organizational factors, and human resources, as well as the limited studies on intrahospital patient transfer in Iran and the lack of comprehensive checklists covering all patient types, this study was designed to develop a comprehensive checklist for safe intrahospital patient transfer in Iranian hospitals and to psychometrically evaluate its properties. Such a culturally appropriate checklist can help improve the safety, reduce adverse events during transfer, lower costs for patients and healthcare facilities, and ultimately improve care quality and patient satisfaction.

## Methods

This study employed a sequential exploratory mixed-methods design [22], which was explicitly planned and conducted in two phases: (1) instrument development and (2) initial validation, which included construct validity analysis. Phase I involved qualitative item generation through a qualitative content analysis of the literature and expert consultations to elicit their tacit professional knowledge. Phase II included an initial assessment of the psychometric properties. The study was conducted in Kashan, Iran, in 2023, with the aim of developing and initially validating a safe intrahospital patient transfer checklist.

### Phase I: Qualitative item generation

The first phase employed a qualitative approach to explore the phenomenon of safe intrahospital transfer and to generate a comprehensive item pool. This phase integrated two complementary data sources to ensure both empirical grounding and clinical relevance, and then integrated the data from these sources.

#### Document analysis

An extensive literature review was conducted as the first method for qualitative data collection. This involved reviewing and analyzing existing literature, clinical guidelines, and institutional protocols related to safe intrahospital patient transfers. The textual content of all relevant and available literature was analyzed using directed qualitative content analysis as described by Assarroudi et al. [23]. This approach enabled structured item generation and categorization of care measures across the pre-transfer, intra-transfer, and post-transfer phases, while remaining open to new, context-specific concepts emerging from the data.

#### Expert elicitation

To complement the document analysis and ground the findings in clinical practice, an expert elicitation process was conducted with a panel of experts consisting of two nursing professors and five clinical nurses (including one nurse, a head nurse, two supervisors, and a senior nurse manager). In line with established qualitative methodologies for eliciting tacit professional knowledge [24, 25], experts were asked to independently list critical care measures necessary for safe intrahospital patient transfer. Their responses were treated as the second source of qualitative data representing lived professional experience. This process allowed us to generate a clinically relevant item pool, effectively capturing the experts’ tacit knowledge and clinical expertise in a structured format.

### Data integration and synthesis

Findings from both sources were synthesized iteratively. The research team compared and integrated items generated by the two aforementioned methods, merged semantically similar statements, refined wording for clarity, and ensured contextual fit. This integration produced a comprehensive, evidence-informed, and experientially grounded pool of checklist items, thereby fulfilling the exploratory qualitative phase of the mixed-methods design. Polit and Beck (2021) emphasized that panels combining academic experts and frontline clinicians are a rigorous approach to qualitative data production and synthesis in scale development, helping to produce items grounded in real-world workflows [24].

### Phase II: Psychometric evaluation

The checklist was originally developed in Persian to ensure cultural and linguistic appropriateness within the Iranian clinical context. The original Persian version of the Safe Intrahospital Patient Transfer Checklist is provided as a supplementary file [Supplementary File 1]. The psychometric evaluation of the checklist was conducted in four steps:

### Step I: Face validity

For the face validity assessment, 10 nursing professors from the Faculty of Nursing at Kashan University of Medical Sciences and 10 nurses from various departments of Shahid-Beheshti Hospital were asked to share their views on the wording, readability, ambiguity, and relevance of each item. Their suggested amendments were implemented accordingly. The same experts were then invited to rate the importance of each item using a five-point Likert scale, ranging from “5: absolutely important” to “1: not important.” After calculating the mean importance score for each item and the frequency of experts rating an item as 4 or 5, the impact scores for the items were calculated using the following formula:

**Item Impact Score= Importance (%)×Frequency**

### Step II: Content validity assessment

In the qualitative content validity assessment, 10 experts (including three nursing professors, two nurses, two head nurses, two supervisors, and a senior nurse manager), reviewed the items and provided comments. After applying their suggested revisions, the experts rated each item as either “Necessary,” “Useful but not necessary,” or “Unnecessary.” The content validity ratio (CVR) for each item was then calculated using the following formula: **CVR = (nE – N/2)/(N/2)**, where *nE* is the number of experts rating the item as “necessary,” and *N* is the total number of experts. According to Lawshe, a CVR of ≥ 0.62 is acceptable when there are ten experts [25].

The content validity index (CVI) was calculated using the Waltz and Bausell method. To this end, the same experts rated the relevance of each item on a 4‑point scale ranging from “1 = Irrelevant” to “4 = Completely relevant”. Items with CVI values > 0.79 were considered appropriate. The CVI for each item was then calculated by dividing the number of experts who rated the item as 3 or 4 by the total number of experts. Items with CVI values < 0.70 were removed, those with CVI values > 0.79 were accepted, and items with CVI values between 0.70 and 0.79 were revised [24, 26].

### Step III: Construct validity and floor and ceiling effects assessment

Exploratory Factor Analysis (EFA) was used to assess the construct validity of the checklist. Confirmatory Factor Analysis (CFA) was not performed, as this study represents the initial development and validation of the instrument, and EFA is recommended at this stage to uncover the underlying factor structure without imposing a rigid, pre-specified model [27, 28]. The theoretical framework of three temporal phases (pre-, during-, and post-transfer) informed the item pool and provided a conceptual starting point for this exploration. Once the factor structure is established in this initial sample, it can be formally tested using CFA in future studies [27, 28].

For EFA, principal component analysis, using the fixed factor method (based on the checklist’s design encompassing three distinct domains: care before, during, and after patient transfer). A varimax rotation was employed. The sample size for this procedure was determined according to the rule of thumb, which typically recommends selecting 10 subjects per item [27]. Floor and ceiling effects were also assessed by determining the rates of participants scoring at the lowest and highest possible extremes of the IHT score range [29].

Since the checklist consisted of 38 items, 380 cases were observed accordingly. Consequently, all IHTs from different wards were monitored until the required sample size was achieved. To minimize the Hawthorne effect—that is, behavioral changes in response to being observed—the nursing services manager introduced the researcher (A.F.) as a permanent member of the IHT team. Subsequently, head nurses informed the researcher about patient transfers in each ward, and the researcher accompanied the transfers from start to finish.

### Step IV: Reliability assessment

This step was performed before the construct validity assessment. To assess the reliability of the checklist, inter-rater reliability was used after two raters independently but simultaneously assessed 30 cases of IHTs. To this end, two nurses were trained to rate each IHT simultaneously but independently using the 38-item checklist. Each item on the checklist was scored on an ordinal scale as follows: 2 = ‘Completely done’, 1 = ‘Incompletely done’, 0 = ‘Not done’ and ‘Not applicable’.” The total checklist score for each rater was calculated by summing the items to produce a continuous total. Reliability was then examined both at the item level and the total-score level using multiple complementary indices. Item-level agreement was quantified using percent agreement and Cohen’s kappa (weighted kappa). For the total score, intraclass correlation coefficients (ICC) with 95% confidence intervals were calculated. Internal consistency across the 38 items was assessed using Cronbach’s alpha [24, 30].

### Statistical analysis

All statistical analyses were performed using the SPSS software version 16.

### Ethical considerations

This study received approval from the Ethics Committee of Kashan University of Medical Sciences (IR.KAUMS.NUHEPM.REC.1397.003). Informed consent was obtained from all participants prior to their involvement. For nurses observed during the reliability check phase, informed consent was obtained after the observation was completed. All participants were briefed on the study’s aims and assured that their data would remain confidential and that the checklists would be anonymized.

## Results

### Development of the checklist

The initial item pool generated from the literature review included 45 items. After the research team reviewed and combined these with the expert panel’s lists, the total increased to 55 items. These 55 items formed the first draft of the checklist.

### Face and content validity assessment

During the face validity assessment, six items were revised, four were merged due to overlaps, and four were deleted because their impact scores were less than 1.5. The remaining 49 items had impact scores greater than 1.5.

In the qualitative content validity assessment, six items were deleted, one item was replaced with a new one, and two were broken into two items. A total of 45 items were examined for their CVR. Five items with CVR values less than 0.62 were removed, while the remaining items had CVRs ranging from 0.9 to 0.98. Next, 40 items underwent CVI evaluation. At this step, two items were removed due to CVIs < 0.70, and 3 items with CVIs between 0.70 and 0.79 were revised [24]. The CVI values of the remaining 38 items ranged from 0.9 to 0.99.

### Construct validity: Exploratory factor analysis

Among the 380 observed IHTs, nurses and nurse assistants performed 43.5% and 32.2% of the transfers, respectively. Other staff members, including orderlies and porters, performed the remaining transfers.

The Kaiser-Meyer-Olkin (KMO) measure of sampling adequacy was 0.916, indicating excellent adequacy for factor analysis. Bartlett’s test of sphericity was significant (χ² = 6598.044, df = 703, *p* < 0.001), confirming the suitability of the data for factor extraction. The principal component analysis extracted three factors, consistent with the theoretical three domains of the checklist. Factors 1 to 3 represented care measure per, during, and post-IHT. The initial eigenvalues for these factors were 11.62, 2.15, and 1.72, accounting for 30.58%, 8.68%, and 7.53% of variance respectively, with a cumulative variance explained of 46.79%. After varimax rotation, the three factors accounted for 46.79% of the total variance, with factor loadings showing clear item clustering within the expected domains. The rotated component matrix demonstrated that items related to care before patient transfer loaded primarily on the first factor, items related to care during transfer loaded on the second factor, and items concerning care after transfer loaded on the third factor, supporting the theoretical structure of the checklist.

In the assessment of floor and ceiling effects, no cases achieved the minimum possible score, while only two cases (0.6%) achieved the maximum score. Therefore, the checklist demonstrates no appreciable floor or ceiling effects.

### Reliability assessment

A total of 38 items were entered into the reliability assessment, of which, no items were removed. The mean percent agreement across the 38 items was 88% (range for individual items: 70%–100%). The mean Cohen’s kappa across items was 0.62 ± 0.12 (range 0.30–0.85; 95% CI: 0.55–0.69). The ICC for the total score was 0.86 (95% CI: 0.74–0.93). Cronbach’s alpha for the entire checklist was 0.79, with subscale values ranging from 0.70 to 0.83.

The remaining 38 items represent care measures to be followed before (15 items), during (10 items), and after (13 items) patient transport. All items are scored on an ordinal scale: 2 (“Completely done”), 1 (“Incompletely done”), or 0 (“Not done” or “Not applicable”). Table 1 presents the CVR, CVI, impact scores, and Factor loading values for these items.

**Table 1:** Items’ content validity, face validity, and factor loading values

No.	Items^1^	CVR^2^	CVI^3^	Impact Score	Factor loading values		
					Factor 1	Factor 2	Factor 3
1	The relevant nurse checked the patient’s level of consciousness before transfer.	1	1	4.9	**0.511**	0.273	0.071
2	The relevant nurse assessed the patient’s respiratory status before transfer (including SpO2 check if necessary).	0.6	0.8	4.9	**0.485**	0.056	0.111
3	The relevant nurse checked the patient’s vital signs (BP, T, PR, and RR) before transfer.	0.83	0.91	4.05	**0.423**	0.254	0.154
4	The relevant nurse verified the physician’s transfer order prior to the transfer.	0.6	0.91	4.9	**0.360**	-0.015	− 0.043
5	The relevant nurse coordinated with the destination department before the transfer.	0.6	0.91	4.9	**0.539**	0.124	− 0.175
6	Hemodynamic disturbances (e.g., abnormal test results) and important events (e.g., respiratory arrest, hypotension, decreased SpO2) were documented in the patient’s file before transfer.	1	1	4.8	**0.605**	0.343	0.140
7	The relevant nurse recorded the patient’s vital signs (BP, T, RR, PR) in the patient’s file before transfer.	0.83	1	4.14	**0.583**	0.098	0.234
8	The patient’s level of consciousness was documented by the nurse before transfer.	0.83	0.91	4.14	**0.521**	0.258	0.210
9	The authorized nurse provided necessary clinical information, including test abnormalities, and significant events, to the receiving department before transfer.	0.6	1	3.36	**0.515**	0.139	0.408
10	The authorized nurse arranged appropriate transport equipment (wheelchair, bed, stretcher) for the patient.	0.83	0.91	4.9	**0.510**	0.108	0.387
11	The authorized nurse entered the patient’s file information, including medication and laboratory tests, into the hospital’s computer system before transfer.	0.83	0.91	4.9	**0.486**	-0.255	-0.002
12	Any administration of blood products or specialized medications (e.g., dopamine, epinephrine) was documented in the patient’s file.	0.83	1	4.9	**0.457**	0.273	0.324
13	Pre-transfer invasive procedures or interventions (e.g., catheter insertion, intubation) were recorded in the file.	0.6	0.91	4.23	**0.593**	-0.009	0.000
14	The relevant nurse provided necessary explanations to the patient or family regarding the reason for transfer.	0.83	1	4.9	**0.607**	0.085	0.311
15	Before the transfer, the nurse ensured the safety of the transfer path, confirming that the patient’s bed and equipment could pass through.	1	1	4.8	**0.483**	0.139	0.175
16	The nurse responsible for the transfer remained with the patient from the beginning of the transport until clinical handover was complete.	1	1	4.9	**0.754**	0.346	0.196
17	The accompanying nurse ensured the bed’s integrity and kept bedsides elevated during transfer to maintain patient’s safety.	0.83	0.91	4.8	0.373	**0.407**	0.067
18	Patient privacy was respected during the transfer process.	1	0.91	4.05	-0.179	**0.441**	0.260
19	The patient was positioned appropriately as prescribed during transfer.	0.6	1	4.6	0.233	**0.462**	0.086
20	Any events occurring during transfer (e.g., apnea, hypotension, decreased SpO2) were recorded in the patient’s file.	0.83	1	4.7	0.216	**0.548**	0.275
21	Necessary equipment was present during transfer depending on the patient’s condition (emergency drugs, monitor, oxygen capsule, ambu bag, CPR bag).	0.83	0.91	4.7	0.091	**0.603**	0.232
22	The nurse took appropriate infection prevention measures during transfer according to the patient’s condition.	0.6	1	4.7	0.367	**0.501**	0.199
23	The relevant nurse ensured the proper functioning of all patient lines and connections (e.g., Shaldon catheter, central venous catheter, peripheral IV lines) during transfer.	1	1	4.9	0.245	**0.622**	0.361
24	Patient’s vital signs were monitored by the nurse during transfer.	0.6	1	4.8	0.219	**0.657**	0.352
25	In emergencies, a nurse trained in CPR accompanies the patient during the transfer.	0.83	0.91	4.9	0.128	**0.634**	0.392
26	The accompanying nurse provided a clinical handover to the receiving nurse at the destination department.	1	0.91	4.8	0.198	**0.690**	0.095
27	The patient’s file was handed over to the receiving nurse.	1	1	4.8	0.134	0.037	**0.482**
28	The medication box and the patient transfer form were handed over to the receiving nurse in the destination.	0.83	0.91	4.9	0.255	-0.381	**0.619**
29	The patient and his/her family received explanations about the new place, its rules, and expectations at the end of transfer.	0.6	0.91	4.9	0.108	0.389	**0.694**
30	The patient’s equipment and personal medications (e.g., insulin pen) were handed over to the receiving nurse, with a receipt obtained.	1	0.91	4.9	0.340	-0.152	**0.588**
31	The patient’s expensive belongings were handed over according to hospital policies (in the presence of the patient, receiving nurse, or security officer) and were recorded in the file.	1	0.91	4.8	0.137	0.231	**0.542**
32	The patient’s nutritional status (oral intake, NPO status, tube feeding, diet type) was reported to the receiving nurse.	0.83	0.91	4.8	0.133	0.029	**0.575**
33	The presence and status of any pressure ulcers (extent, degree, location) were communicated to the receiving nurse.	0.6	1	**4.8**	0.138	0.224	**0.439**
34	The status of patient connections (e.g., Shaldon catheter, CVC, IV lines) was reported to the receiving nurse.	0.6	1	**4.9**	0.160	0.199	**0.652**
35	Any complications related to the patient’s lines (e.g., phlebitis) were reported to the receiving nurse.	083	0.91	**4.9**	0.144	0.415	**0.677**
36	The condition of any bandaged areas (chest tube sites, drains, wounds) was reported to the receiving nurse.	1	1	**4.9**	0.165	0.404	**0.607**
37	The patient’s therapeutic, diagnostic, and delayed care plans were reported to the receiving nurse.	1	1	**4.9**	0.158	0.370	**0.626**
38	The patient handover form was signed by the receiving nurse.	0.83	0.91	**4.9**	-0.002	0.063	**0.421**

^1^Items 1–15 assess the pretransfer phase, items 16–25 assess the intra-transfer phase, 26–38 assess the post-transfer phase, ^2^Content Validity Ratio, ^3^Content Validity Index to be followed before (15 items), during (10 items), and after (13 items) patient transport

## Discussion

Research has consistently shown that intrahospital patient transfers are often poorly executed, endangering patient safety [9, 31, 32]. Key factors contributing to this include inadequate staffing, lack of experience, insufficient knowledge, ineffective communication, and faulty equipment [33]. Furthermore, current training programs are typically insufficient, leaving medical staff inadequately prepared for safe transfers. Shortcomings in IHTs are linked to high rates of adverse events, so that over 30% of patients’ experience complications during transport. Implementing structured protocols has been proven to drastically reduce these incidents [34]. Consequently, organizations like The Joint Commission of National Patient Safety Goals recommend establishing standardized protocols to improve communication and safety [35]. Developing standardized checklists along with continuous training can enhance IHT safety, establish clear guidelines, improve team communication, ensure equipment is functional, and help mitigate risks [36, 37]. They also empower nurses to make timely, informed decisions [20, 38]. However, a checklist alone is ineffective; its success depends on integration with education, organizational support, supervision, and adequate resources [39]. Addressing this critical need, our study developed and validated a comprehensive checklist for safe IHT, covering essential measures across the pre-transfer, during-transfer, and post-transfer phases.

The development process followed evidence-based guidelines. An initial item pool was generated through a systematic literature review and refined by inputs from an expert panel, ensuring content relevance and comprehensiveness [24, 40]. Iterative steps, including face validity assessment and qualitative content validation, decreased the items, and enhanced clarity and usability [26]. Quantitative content validity measures (CVR and CVI) further supported item essentiality and validity, applying established thresholds (CVR ≥ 0.62, CVI ≥ 0.70) [41]. These steps align with methodologies used in similar studies on healthcare safety checklists. For instance, Brunsveld-Reinders et al. (2015) used comparable steps involving literature review, expert input, and iterative checks for their IHTs safety checklist [16] .

Construct validity analysis provided additional support. Bartlett’s test and high KMO values confirmed data adequacy for factor analysis [24]. Principal component analysis revealed a three-factor structure corresponding to pre-, during-, and post-transfer phases, which explained 46% of the total variance in transfer safety. Each factor contributed between 7% and 30% of the variance, meeting recommended psychometric standards for multidimensional instruments [42].

Moreover, the checklist demonstrated no significant floor or ceiling effects, indicating that it is composed of appropriate items to assess the safety of IHTs. A checklist is considered to have these effects if more than 15% of cases achieve either the minimum or maximum possible score [29].

Reliability analyses further confirmed the checklist’s robustness. Interrater reliability, measured by Cohen’s kappa (0.62), indicated moderate-to-substantial agreement, while the ICC of 0.86 demonstrated excellent reliability. Internal consistency was also strong, with Cronbach’s alpha values of 0.79 overall and 0.70–0.83 across subscales, in line with accepted benchmarks [43].

Taken together, these findings validate our checklist as a reliable and valid tool for capturing the multidimensional aspects of safe intrahospital patient transfer. Its methodological rigor parallels other well-established healthcare checklists, supporting its practical application for improving patient safety.

This is the first checklist in Iran developed for the intrahospital transfer of a broad range of patients. A key strength of this study is its exploratory sequential mixed-methods design, which began with a qualitative phase that combined document analysis and expert elicitation to identify context-specific care measures for safe patient transfer and continued with psychometric evaluation, including EFA. This approach ensured that the resulting instrument was conceptually grounded in both the global evidence base and local clinical realities. Although it has demonstrated strong psychometric properties, it is not without limitations. While expert consultations provided rich qualitative input, future studies could include patient or frontline staff interviews to incorporate additional. Widespread implementation may also reveal areas for further refinement. CFA was not performed in this study because it represents the first development and validation of the instrument. Once the structure is established and replicated in future samples, CFA can then be conducted for confirmation. Following its development, the checklist was approved by the Ministry of Health and Medical Education for use across Iranian hospitals. For successful adoption, however, focused education, ongoing support, and supervision of healthcare teams—particularly nurses—will be essential. Future studies should also evaluate its impact on patient safety outcomes in practice.

## Conclusion

Our study developed and psychometrically validated a comprehensive checklist for safe intrahospital patient transfer. By providing structured guidance across pre-, during-, and post-transfer phases, the checklist has the potential to improve transfer quality, enhance patient safety, and support healthcare professionals in clinical decision-making. Its widespread use, accompanied by appropriate training and organizational support, can contribute to reducing adverse events and improving patient care during transfers.

## Supplementary Information

Below is the link to the electronic supplementary material.

Supplementary Material 1

Supplementary Material 2

## Data Availability

Data set of this study will be available from the corresponding author for reasonable reasons.

## Abbreviations

CVR: Content validity ratio
CVI: Content validity index
IHT: Intrahospital transfer
ICC: Intraclass correlation coefficients
EFA: Exploratory Factor Analysis
CFA: Confirmatory Factor Analysis
