# Supplementary material for: Developing and psychometrics assessment of a checklist for safe intrahospital patient transfer
Source: BMC Health Serv Res. 2026 Jan 8;26:185. doi: 10.1186/s12913-025-13987-w (PMC12882135; doi:10.1186/s12913-025-13987-w)
Supplement: Supplementary file 1 — Supplementary Material 1 [file 12913_2025_13987_MOESM1_ESM.docx]

**Checklist for intra hospital patient transfer**

*** ***

**Purpose of this Checklist:**
This checklist is a standardized tool designed to ensure patient safety, continuity of care, and effective communication during the transfer of a patient from one hospital department to another. It guides healthcare providers through the critical steps in the **Pre-transfer**, **Intra-transfer**, and **Post-transfer** phases to minimize risks and prevent errors.

The primary user is the **nurse** responsible for coordinating and executing the patient transfer.

**How to Use This Checklist:**

1. **Complete the Header:** Before starting, fill in all patient and transfer details at the top of the form (Patient sex, destination, origin, cause of transfer, GCS, transfer vehicle, and accompanying personnel).
2. **Performance Rating:** For each item, check the box that best reflects the completion status:
   - **Completely Done:** The action was fully and correctly performed as described.
   - **Incompletely Done:** The action was started but not finished, or key elements were missed.
   - **Not Done:** The required action was not performed.
   - **Not Applicable:** Use if the item does not pertain to this specific patient's condition or situation.

*** ***

Patient sex: Male 󠆞🞎 Female 🞎, Destination ward: ………….., Originating ward:………..

Cause of transfer:……………… GCS of patient: ………….,

Transfer vehicle: Patient’s bed 🞎 Wheelchair 🞎 Stretcher 🞎 By patient’s feet 🞎

Who accompany during transfer: Nurse 🞎 Nurse assistant 🞎 An orderly 🞎 Relatives 🞎

**Checklist for intra hospital patient transfer**

| **Performance rating** | | | | **Items** |  |
| --- | --- | --- | --- | --- | --- |
| Not applicable | Not done | Incompletely done | Completely done | The relevant nurse checked the patient’s level of consciousness before transfer. | Pre-transfer care measures |
| Not applicable | Not done | Incompletely done | Completely done | The relevant nurse assessed the patient’s respiratory status before transfer (including SpO2 check if necessary). |  |
| Not applicable | Not done | Incompletely done | Completely done | The relevant nurse checked the patient’s vital signs (BP, T, PR, and RR) before transfer. |  |
| Not applicable | Not done | Incompletely done | Completely done | The relevant nurse verified the physician’s transfer order prior to the transfer. |  |
| Not applicable | Not done | Incompletely done | Completely done | The relevant nurse coordinated with the destination department before the transfer. |  |
| Not applicable | Not done | Incompletely done | Completely done | Hemodynamic disturbances (e.g., abnormal test results) and important events (e.g., respiratory arrest, hypotension, decreased SpO2) were documented in the patient’s file before transfer. |  |
| Not applicable | Not done | Incompletely done | Completely done | The relevant nurse recorded the patient’s vital signs (BP, T, RR, PR) in the patient’s file before transfer. |  |
| Not applicable | Not done | Incompletely done | Completely done | The patient's level of consciousness was documented by the nurse before transfer. |  |
| Not applicable | Not done | Incompletely done | Completely done | The authorized nurse provided necessary clinical information, including test abnormalities, and significant events, to the receiving department before transfer. |  |
| Not applicable | Not done | Incompletely done | Completely done | The authorized nurse arranged appropriate transport equipment (wheelchair, bed, stretcher) for the patient. |  |
| Not applicable | Not done | Incompletely done | Completely done | The authorized nurse entered the patient's file information, including medication and laboratory tests, into the hospital's computer system before transfer. |  |
| Not applicable | Not done | Incompletely done | Completely done | Any administration of blood products or specialized medications (e.g., dopamine, epinephrine) was documented in the patient’s file. |  |
| Not applicable | Not done | Incompletely done | Completely done | Pre-transfer invasive procedures or interventions (e.g., catheter insertion, intubation) were recorded in the file. |  |
| Not applicable | Not done | Incompletely done | Completely done | The relevant nurse provided necessary explanations to the patient or family regarding the reason for transfer. |  |
| Not applicable | Not done | Incompletely done | Completely done | Before the transfer, the nurse ensured the safety of the transfer path, confirming that the patient's bed and equipment could pass through. |  |
| Not applicable | Not done | Incompletely done | Completely done | The nurse responsible for the transfer remained with the patient from the beginning of the transport until clinical handover was complete. | Intra-transfer care measures |
| Not applicable | Not done | Incompletely done | Completely done | The accompanying nurse ensured the bed’s integrity and kept bedsides elevated during transfer to maintain patient's safety. |  |
| Not applicable | Not done | Incompletely done | Completely done | Patient privacy was respected during the transfer process. |  |
| Not applicable | Not done | Incompletely done | Completely done | The patient was positioned appropriately as prescribed during transfer. |  |
| Not applicable | Not done | Incompletely done | Completely done | Any events occurring during transfer (e.g., apnea, hypotension, decreased SpO2) were recorded in the patient’s file. |  |
| Not applicable | Not done | Incompletely done | Completely done | Necessary equipment was present during transfer depending on the patient's condition (emergency drugs, monitor, oxygen capsule, ambu bag, CPR bag). |  |
| Not applicable | Not done | Incompletely done | Completely done | The nurse took appropriate infection prevention measures during transfer according to the patient’s condition. |  |
| Not applicable | Not done | Incompletely done | Completely done | The relevant nurse ensured the proper functioning of all patient lines and connections (e.g., Shaldon catheter, central venous catheter, peripheral IV lines) during transfer. |  |
| Not applicable | Not done | Incompletely done | Completely done | Patient's vital signs were monitored by the nurse during transfer. |  |
| Not applicable | Not done | Incompletely done | Completely done | In emergencies, a nurse trained in CPR accompanies the patient during the transfer. |  |
| Not applicable | Not done | Incompletely done | Completely done | The accompanying nurse provided a clinical handover to the receiving nurse at the destination department. | Post-transfer care measures |
| Not applicable | Not done | Incompletely done | Completely done | The patient's file was handed over to the receiving nurse. |  |
| Not applicable | Not done | Incompletely done | Completely done | The medication box and the patient transfer form were handed over to the receiving nurse in the destination. |  |
| Not applicable | Not done | Incompletely done | Completely done | The patient and his/her family received explanations about the new place, its rules, and expectations at the end of transfer. |  |
| Not applicable | Not done | Incompletely done | Completely done | The patient's equipment and personal medications (e.g., insulin pen) were handed over to the receiving nurse, with a receipt obtained. |  |
| Not applicable | Not done | Incompletely done | Completely done | The patient's expensive belongings were handed over according to hospital policies (in the presence of the patient, receiving nurse, or security officer) and were recorded in the file. |  |
| Not applicable | Not done | Incompletely done | Completely done | The patient's nutritional status (oral intake, NPO status, tube feeding, diet type) was reported to the receiving nurse. |  |
| Not applicable | Not done | Incompletely done | Completely done | The presence and status of any pressure ulcers (extent, degree, location) were communicated to the receiving nurse. |  |
| Not applicable | Not done | Incompletely done | Completely done | The status of patient connections (e.g., Shaldon catheter, CVC, IV lines) was reported to the receiving nurse. |  |
| Not applicable | Not done | Incompletely done | Completely done | Any complications related to the patient's lines (e.g., phlebitis) were reported to the receiving nurse. |  |
| Not applicable | Not done | Incompletely done | Completely done | The condition of any bandaged areas (chest tube sites, drains, wounds) was reported to the receiving nurse. |  |
| Not applicable | Not done | Incompletely done | Completely done | The patient's therapeutic, diagnostic, and delayed care plans were reported to the receiving nurse. |  |
| Not applicable | Not done | Incompletely done | Completely done | The patient handover form was signed by the receiving nurse. |  |
