# Supplementary material for: Developing and psychometrics assessment of a checklist for safe intrahospital patient transfer
Source: BMC Health Serv Res. 2026 Jan 8;26:185. doi: 10.1186/s12913-025-13987-w (PMC12882135; doi:10.1186/s12913-025-13987-w)
Supplement: Supplementary file 2 — Supplementary Material 2 [file 12913_2025_13987_MOESM2_ESM.docx]

**چک لیست انتقال ایمن داخل بیمارستانی بیماران**

*** ***

**هدف از این چک لیست:**

**این چک لیست ابزاری استاندارد است که برای اطمینان از ایمنی بیمار، تداوم مراقبت و ارتباط موثر در طول انتقال بیمار از یک بخش بیمارستان به بخش دیگر طراحی شده است. این چک لیست، ارائه دهندگان خدمات درمانی را در مراحل حساس قبل از انتقال، حین انتقال و پس از انتقال راهنمایی می‌کند تا خطرات را به حداقل رسانده و از خطاها جلوگیری کند.**

**کاربر اصلی، پرستاری است که مسئول هماهنگی و اجرای انتقال بیمار است.**

**نحوه استفاده از این چک لیست:**

**1. تکمیل سربرگ: قبل از شروع، تمام جزئیات بیمار و انتقال را که در بالای فرم ذکر شده اند پر کنید (جنسیت بیمار، مقصد، مبدا، علت انتقال، GCS، وسیله نقلیه انتقال و پرسنل همراه).**

**2. نحوه رتبه بندی عملکرد: برای هر مورد، کادری را که به بهترین وجه وضعیت انجام مراقبت را نشان می‌دهد، علامت بزنید:**

**- بصورت کامل انجام شد: عمل به طور کامل و صحیح مطابق توضیحات انجام شده است.**

**- بصورت ناقص انجام شد: عمل شروع شده اما تمام نشده است یا عناصر کلیدی از قلم افتاده‌اند.**

**- انجام نشده: عمل مورد نیاز انجام نشده است.**

**- لازم نبود: این گزینه در صورتی انتخاب شود که مورد به شرایط یا وضعیت خاص این بیمار مربوط نبوده و لازم نبوده است.**

*** ***

**جنسیت بیمار: مرد** 🞎 **زن** 🞎**، بخش مقصد: …………..، بخش مبدا: ..................**

**علت انتقال: ………………، GCS بیمار: ………….،**

**وسیله نقلیه انتقال: تخت بیمار** 🞎 **ویلچر** 🞎  **برانکارد** 🞎  **راه رفتن بیمار با پای خود** 🞎

**فردی که در طول انتقال همراه بیمار است: پرستار** 🞎 **کمک پرستار** 🞎 **یک نفر از پرسنل** 🞎 **بستگان بیمار** 🞎

**چک لیست انتقال ایمن داخل بیمارستانی بیماران**

| No**.** | **گویه** | بصورت کامل انجام شد | بصورت ناقص انجام شد | انجام نشد | لازم نبود |
| --- | --- | --- | --- | --- | --- |
| اقدامات پیش از انتقال | قبل از انتقال، پرستار مسئول سطح هوشیاری بیمار را بررسی کرد | بصورت کامل انجام شد | بصورت ناقص انجام شد | انجام نشد | لازم نبود |
|  | قبل از انتقال، پرستار مسئول وضعیت تنفسی (شامل بررسی SpO2 در صورت لزوم) بیمار را ارزیابی کرد. | بصورت کامل انجام شد | بصورت ناقص انجام شد | انجام نشد | لازم نبود |
|  | قبل از انتقال، پرستار مسئول علائم حیاتی بیمار (فشار خون، دما، نبض، و تعداد تنفس) را بررسی کرد. | بصورت کامل انجام شد | بصورت ناقص انجام شد | انجام نشد | لازم نبود |
|  | قبل از انتقال، پرستار مسئول، دستور انتقال صادر شده توسط پزشک را تأیید کرد. | بصورت کامل انجام شد | بصورت ناقص انجام شد | انجام نشد | لازم نبود |
|  | قبل از انتقال، پرستار مسئول با بخش مقصد هماهنگی نمود. | بصورت کامل انجام شد | بصورت ناقص انجام شد | انجام نشد | لازم نبود |
|  | قبل از انتقال، اختلالات همودینامیک (مثل نتایج غیرطبیعی آزمایشات) و رویدادهای مهم (مثل ایست تنفسی، افت فشار خون، کاهشSpO2) در پرونده بیمار ثبت شد | بصورت کامل انجام شد | بصورت ناقص انجام شد | انجام نشد | لازم نبود |
|  | قبل از انتقال، پرستار مسئول علائم حیاتی بیمار (فشار خون، دما، تعداد تنفس، نبض) را در پرونده بیمار ثبت کرد. | بصورت کامل انجام شد | بصورت ناقص انجام شد | انجام نشد | لازم نبود |
|  | قبل از انتقال، پرستار مسئول سطح هوشیاری بیمار را در پرونده ثبت کرد. | بصورت کامل انجام شد | بصورت ناقص انجام شد | انجام نشد | لازم نبود |
|  | قبل از انتقال، پرستار مسئول، اطلاعات بالینی لازم از جمله نتایج غیرطبیعی آزمایش‌ها و رویدادهای مهم را به بخش مقصد ارائه داد. | بصورت کامل انجام شد | بصورت ناقص انجام شد | انجام نشد | لازم نبود |
|  | پرستار مسئول، تجهیزات انتقال مناسب (صندلی چرخدار، تخت، برانکارد) را برای بیمار فراهم کرد. | بصورت کامل انجام شد | بصورت ناقص انجام شد | انجام نشد | لازم نبود |
|  | قبل از انتقال، پرستار مسئول، اطلاعات پرونده بیمار از جمله داروها و تستهای آزمایشگاهی را در سیستم کامپیوتری بیمارستان وارد کرد. | بصورت کامل انجام شد | بصورت ناقص انجام شد | انجام نشد | لازم نبود |
|  | قبل از انتقال، تجویز هرگونه فرآورده‌ خونی یا داروهای تخصصی (مانند دوپامین، اپینفرین)، در پرونده بیمار ثبت شد. | بصورت کامل انجام شد | بصورت ناقص انجام شد | انجام نشد | لازم نبود |
|  | قبل از انتقال، اقدامات یا مداخلات تهاجمی (مانند گذاشتن کاتتر، لوله گذاری تراشه) در پرونده ثبت شد. | بصورت کامل انجام شد | بصورت ناقص انجام شد | انجام نشد | لازم نبود |
|  | پرستار مسئول، توضیحات لازم را در مورد دلیل انتقال، به بیمار یا خانواده وی ارائه داد. | بصورت کامل انجام شد | بصورت ناقص انجام شد | انجام نشد | لازم نبود |
|  | قبل از انتقال، پرستار از ایمنی مسیر انتقال اطمینان حاصل کرد و مطمئن شد که تخت و تجهیزات بیمار قابلیت عبور از مسیر را دارند. | بصورت کامل انجام شد | بصورت ناقص انجام شد | انجام نشد | لازم نبود |
| اقدامات حین انتقال | پرستار مسئول انتقال، از شروع جابجایی تا تکمیل تحویل بالینی، همراه بیمار باقی ماند. | بصورت کامل انجام شد | بصورت ناقص انجام شد | انجام نشد | لازم نبود |
|  | پرستار همراه، از سالم بودن تخت اطمینان حاصل کرد و نرده کنار تخت را در طول انتقال بالا نگه داشت تا ایمنی بیمار حفظ شود. | بصورت کامل انجام شد | بصورت ناقص انجام شد | انجام نشد | لازم نبود |
|  | در طول فرآیند انتقال، حریم خصوصی بیمار رعایت شد. | بصورت کامل انجام شد | بصورت ناقص انجام شد | انجام نشد | لازم نبود |
|  | در حین انتقال، بیمار در پوزیشن مناسب و طبق دستور پزشک قرار داده شد. | بصورت کامل انجام شد | بصورت ناقص انجام شد | انجام نشد | لازم نبود |
|  | هرگونه رویداد رخ داده در حین انتقال (مانند آپنه، افت فشار خون، کاهشSpO2) در پرونده بیمار ثبت شد. | بصورت کامل انجام شد | بصورت ناقص انجام شد | انجام نشد | لازم نبود |
|  | در حین انتقال، تجهیزات لازم (مانند داروهای اورژانسی، مانیتور، کپسول اکسیژن، آمبوبگ، کیف احیا) با توجه به وضعیت بیمار موجود بود. | بصورت کامل انجام شد | بصورت ناقص انجام شد | انجام نشد | لازم نبود |
|  | پرستار در طول انتقال، با توجه به وضعیت بیمار، اقدامات پیشگیرانه مناسب جهت جلوگیری از عفونت را انجام داد. | بصورت کامل انجام شد | بصورت ناقص انجام شد | انجام نشد | لازم نبود |
|  | در طول انتقال، پرستار مسئول از عملکرد صحیح تمامی لاین‌ها و اتصالات بیمار (مانند کاتتر شلدون، کاتتر ورید مرکزی، لاین‌های وریدی محیطی) اطمینان حاصل کرد. | بصورت کامل انجام شد | بصورت ناقص انجام شد | انجام نشد | لازم نبود |
|  | در طول انتقال، علائم حیاتی بیمار توسط پرستار پایش شد. | بصورت کامل انجام شد | بصورت ناقص انجام شد | انجام نشد | لازم نبود |
|  | در شرایط اورژانسی، یک پرستار آموزش دیده در زمینه احیای قلبی ریوی (CPR) بیمار را در حین انتقال همراهی می‌کند. | بصورت کامل انجام شد | بصورت ناقص انجام شد | انجام نشد | لازم نبود |
| اقدامات پس از انتقال | در بخش مقصد، پرستار همراه، بیمار را بصورت بالینی به پرستار بخش جدید تحویل داد. | بصورت کامل انجام شد | بصورت ناقص انجام شد | انجام نشد | لازم نبود |
|  | در بخش جدید، پرونده بیمار به پرستار تحویل گیرنده تحویل داده شد | بصورت کامل انجام شد | بصورت ناقص انجام شد | انجام نشد | لازم نبود |
|  | داروها و فرم انتقال بیمار به پرستار تحویل گیرنده در بخش مقصد تحویل داده شد | بصورت کامل انجام شد | بصورت ناقص انجام شد | انجام نشد | لازم نبود |
|  | در پایان انتقال، به بیمار و خانواده وی در مورد محل جدید، مقررات آن و انتظارات توضیح داده شد | بصورت کامل انجام شد | بصورت ناقص انجام شد | انجام نشد | لازم نبود |
|  | تجهیزات و داروهای شخصی بیمار (مانند قلم انسولین) به پرستار تحویل گیرنده در بخش جدید تحویل داده شد و رسید دریافت گردید. | بصورت کامل انجام شد | بصورت ناقص انجام شد | انجام نشد | لازم نبود |
|  | وسایل قیمتی بیمار طبق سیاست‌های بیمارستان (در حضور بیمار، پرستار تحویل گیرنده یا مأمور امنیتی) تحویل داده شد و در پرونده ثبت گردید. | بصورت کامل انجام شد | بصورت ناقص انجام شد | انجام نشد | لازم نبود |
|  | وضعیت تغذیه بیمار (مقدار مصرف خوراکی، NPO بودن، تغذیه با لوله، نوع رژیم غذایی) به پرستار تحویل گیرنده گزارش شد. | بصورت کامل انجام شد | بصورت ناقص انجام شد | انجام نشد | لازم نبود |
|  | وجود و وضعیت هرگونه زخم فشاری (وسعت، درجه، محل) به پرستار تحویل گیرنده اطلاع داده شد. | بصورت کامل انجام شد | بصورت ناقص انجام شد | انجام نشد | لازم نبود |
|  | وضعیت اتصالات بیمار (مانند کاتتر شلدون، کاتتر ورید مرکزی، لاین‌های وریدی) به پرستار تحویل گیرنده گزارش شد. | بصورت کامل انجام شد | بصورت ناقص انجام شد | انجام نشد | لازم نبود |
|  | هرگونه عارضه مرتبط با لاین‌های بیمار (مانند فلبیت) به پرستار تحویل گیرنده گزارش شد. | بصورت کامل انجام شد | بصورت ناقص انجام شد | انجام نشد | لازم نبود |
|  | وضعیت نواحی پانسمان شده (محل لوله قفسه سینه، درن‌ها، زخم‌ها) به پرستار تحویل گیرنده گزارش شد. | بصورت کامل انجام شد | بصورت ناقص انجام شد | انجام نشد | لازم نبود |
|  | برنامه‌های درمانی، تشخیصی و مراقبتی معوق مانده بیمار به پرستار تحویل گیرنده گزارش شد. | بصورت کامل انجام شد | بصورت ناقص انجام شد | انجام نشد | لازم نبود |
|  | فرم تحویل بیمار توسط پرستار تحویل گیرنده امضا شد. | بصورت کامل انجام شد | بصورت ناقص انجام شد | انجام نشد | لازم نبود |
